# Comparison of Erlotinib vs. Osimertinib for Advanced or Metastatic EGFR Mutation-Positive Non-Small-Cell Lung Cancer Without Prior Treatment: A Network Meta-Analysis

**DOI:** 10.3390/cancers17111895

**Published:** 2025-06-05

**Authors:** Fernando M. Runzer-Colmenares, Rossana Ruiz, Lorenzo Maco, Mike Maldonado, Luis Puma-Villanueva, Marco Galvez-Nino, Carlos Aliaga, Vicente A. Benites-Zapata, Carlos Diaz-Arocutipa, Luis Mas, Diego Urrunaga-Pastor

**Affiliations:** 1 CHANGE Research Working Group, Carrera de Medicina Humana, Universidad Científica del Sur, Lima 15067, Peru; frunzer@cientifica.edu.pe; 2 Departamento de Oncología Médica, Instituto Nacional de Enfermedades Neoplásicas, Lima 15023, Peru; rossana.ruiz@auna.org (R.R.); marco.galvez.n@upch.pe (M.G.-N.); luis.mas.l@upch.pe (L.M.); 3 Departamento de Medicina Oncológica, Oncosalud—AUNA, Lima 15036, Peru; 4 Facultad de Medicina Humana, Universidad Ricardo Palma, Lima 15039, Peru; lorenzo.maco@upch.pe; 5 Departamento de Medicina Oncológica, Hospital Nacional PNP “Luis N. Saenz”, Lima 15048, Peru; 6 Departamento de Oncología Médica, Instituto Regional de Enfermedades Neoplásicas “Dr. Luis Pinillos Ganoza”, Trujillo 13007, Peru; mmaldonadom@irennorte.gob.pe; 7 Servicio de Oncología Médica, Hospital Carlos Alberto Seguín Escobedo EsSalud, Arequipa 04510, Peru; lpuma@ucsm.edu.pe; 8 Facultad de Medicina Humana, Universidad Católica de Santa María, Arequipa 04013, Peru; 9 Facultad de Medicina, Universidad Peruana Cayetano Heredia, Lima 15102, Peru; 10 Servicio de Oncología, Centro Oncológico Aliada, Lima 15036, Peru; carlos.aliaga@aliada.com.pe; 11 Unidad de Investigación para la Generación y Síntesis de Evidencias en Salud, Universidad San Ignacio de Loyola, Lima 15026, Peru; vbenites@usil.edu.pe; 12 Unidad de Revisiones Sistemáticas y Meta-análisis (URSIGET), Vicerrectorado de Investigación, Universidad San Ignacio de Loyola, Lima 15026, Peru; cdiazar@usil.edu.pe

**Keywords:** non-small-cell lung carcinoma, EGFR mutations, osimertinib, erlotinib, network meta-analysis, lung cancer

## Abstract

Non-small cell lung cancer (NSCLC) accounts for 85% of lung cancer cases globally and most patients receive their diagnosis at advanced or metastatic disease stages. The use of tyrosine kinase inhibitors (TKIs) such as erlotinib (first-generation) and osimertinib (third-generation) to treat NSCLC is possible because of activating mutations in the epidermal growth factor receptor (EGFR). Although osimertinib has shown better results in recent trials, direct and updated comparisons with erlotinib, especially in combination regimens, are still limited. **Background/Objectives**: This study aimed to compare the efficacy and safety of osimertinib versus erlotinib, both as monotherapies and in combination, in treatment-naïve patients with advanced or metastatic EGFR-mutated NSCLC. **Methods**: A systematic review and network meta-analysis were conducted following PRISMA-NMA guidelines and registered in PROSPERO (CRD42025649761). PubMed, EMBASE, and Scopus were searched up to February 2025 for randomized controlled trials (RCTs) that compared erlotinib- or osimertinib-based regimens in previously untreated EGFR-mutated advanced NSCLC. Outcomes included overall survival (OS), progression-free survival (PFS), and grade ≥ 3 adverse events. A frequentist random-effects model was used, and treatments were ranked using p-scores. **Results**: Eleven RCTs (2341 patients) were included. Osimertinib, alone or with chemotherapy, resulted in significantly longer OS compared to erlotinib-based regimens (HR for OS vs. erlotinib: 1.59, 95% CI 1.09–2.31). All osimertinib and erlotinib regimens outperformed chemotherapy for PFS, but no statistically significant differences were observed between osimertinib and erlotinib. Severe adverse events were comparable, though osimertinib ranked highest for safety. The combination of osimertinib with chemotherapy achieved the highest p-scores for both OS and PFS. **Conclusions**: Osimertinib is associated with superior overall survival and comparable safety versus erlotinib-based strategies in first-line treatment of advanced EGFR-mutated NSCLC. These findings reinforce osimertinib as the preferred first-line option in this setting.

## Simple Summary

Lung cancer is one of the leading causes of cancer deaths worldwide. Among patients with advanced stages of this disease, some have specific genetic changes that allow for targeted treatments. Two of these treatments, osimertinib and erlotinib, are widely used. However, it remains unclear which one provides better survival and fewer side effects when used alone or in combination with other therapies. This study compares these treatments by analyzing data from several clinical trials. Our findings suggest that osimertinib, especially when combined with chemotherapy, offers better survival outcomes and is generally safer than erlotinib-based treatments. These results provide updated evidence to help doctors choose the most effective first-line treatment for patients with this type of lung cancer, aiming to improve their survival and quality of life.

## 1. Introduction

According to the most recent global cancer statistics, lung cancer remains the leading cause of cancer-related mortality worldwide, underscoring the critical need for effective treatments [1]. Non-small cell lung cancer (NSCLC) accounts for approximately 85% of all lung cancer cases worldwide, with a substantial proportion diagnosed at advanced or metastatic stages, contributing to a poor five-year survival rate estimated at around 26% [2,3,4]. In patients with advanced non-squamous NSCLC, activating mutations in the epidermal growth factor receptor (EGFR)—particularly exon 19 deletions and exon 21 L858R substitutions—constitute key therapeutic targets [5,6]. These alterations are present in approximately 10–15% of Western populations and up to 40–60% of Asian populations and have driven the development of tyrosine kinase inhibitors (TKIs), which have revolutionized the first-line treatment of this malignancy [7,8]. In Perú, EGFR mutations occur in 32% to 39% of patients among all subtypes [9].

Erlotinib, a first-generation TKI, has shown significant clinical benefit over standard chemotherapy in patients harboring sensitizing EGFR mutations [10,11]. However, its efficacy is limited by the emergence of acquired resistance, most notably the T790M mutation [12]. In response, osimertinib—a third-generation TKI—has emerged as a highly effective and well-tolerated alternative, both in pretreated patients and as a first-line therapy, with favorable outcomes reported in trials such as FLAURA and FLAURA-2 [13,14,15].

Several meta-analyses and systematic reviews have sought to synthesize the comparative evidence on TKI use in previously untreated EGFR-mutated advanced or metastatic NSCLC [16]. For instance, Holleman et al. conducted a network meta-analysis including 13 RCTs published up to 2018, identifying osimertinib as the most effective option in terms of progression-free survival (PFS) and with a better toxicity profile compared to other targeted therapies [17]. Similarly, Zhang et al. demonstrated a clinical advantage of osimertinib and TKI–chemotherapy combinations over monotherapy in terms of overall survival (OS) and adverse events [16]. However, these studies excluded newer combination strategies, such as osimertinib with bevacizumab or chemotherapy, and their systematic searches predate 2020, failing to incorporate more recent trials and updated safety data.

A more recent network meta-analysis by Zhang and Sun (2025), which included 30 RCTs and 19 therapeutic strategies up to May 2024, confirmed the superiority of osimertinib—particularly in combination with chemotherapy—in terms of PFS and OS [18]. Nevertheless, their analysis focused on broad comparisons and did not provide a specific evaluation of erlotinib versus osimertinib across monotherapy and combination regimens, considering that erlotinib is one of the most widely available EGFR-TKIs [19]. Additionally, the study did not assess the magnitude of clinical benefit through specific treatment rankings, such as those derived from p-scores.

Therefore, critical gaps remain in the literature regarding head-to-head and updated comparisons between osimertinib and erlotinib, the simultaneous evaluation of efficacy and safety using a consistent methodological framework; and the incorporation of treatment ranking approaches to guide clinical decision-making based on OS, PFS, and toxicity.

To address these limitations, the present study aimed to compare the efficacy and safety of osimertinib (alone or in combination) versus erlotinib (alone or in combination) in treatment-naïve adult patients with EGFR mutation-positive advanced or metastatic NSCLC through a comprehensive network meta-analysis.

## 2. Materials and Methods

This systematic review was reported according to the PRISMA for Network Meta-Analyses (Preferred Reporting Items for Systematic Reviews and Meta-Analyses) statement and registered in the PROSPERO platform (ID: CRD42025649761).

### 2.1. Search Strategy

The following electronic databases were searched from inception to 9 February 2025: PubMed-MEDLINE, EMBASE, and Scopus. There were no date or language restrictions. We also performed a forward and backward citation search of reference lists of included studies to identify other potentially eligible studies. The detailed search strategy is available in Table S1.

### 2.2. Eligibility Criteria

The inclusion criteria were as follows: (i) randomized clinical trials (RCTs) that have used erlotinib or osimertinib and that used chemotherapy or another tyrosine kinase inhibitor as a comparator, (ii) enrolled patients with epidermal growth factor receptor (EGFR) mutation-positive non-small-cell lung cancer without prior treatment, (iii) enrolled adult patients (≥18 years old), and (iv) reported at least one of the assessed outcomes at any length of follow-up. Case reports, case series, observational studies, conference abstracts, reviews, and editorials were excluded.

### 2.3. Study Selection

All articles from the electronic search were downloaded into EndNote 20TM and duplicate records were removed. All unique articles were uploaded to Rayyan (https://rayyan.qcri.org/, accessed 9 February 2025) for the study selection process. Two review authors independently screened the titles/abstracts and full texts to identify relevant studies and registered reasons for exclusions. All discrepancies were resolved by consensus.

### 2.4. Outcomes

The study outcomes were overall survival, progression-free survival, and grade ≥ 3 adverse events. Trial definitions were used for all outcomes.

### 2.5. Data Extraction

We extracted the following information using a standardized data extraction form that was previously piloted: first author name, publication year, country, type of RCT, description of intervention and control group, sample size, age, sex, stage, histologic type, EGFR-mutation type, time of follow-up, and all outcomes per strategy arm.

### 2.6. Risk of Bias Assessment

We used the Cochrane Risk of Bias (RoB) tool 2.0 to assess the risk of bias for each RCT. This tool assesses the following five domains: randomization process, deviations from intended interventions, missing outcome data, measurement of the outcome, and selection of the reported result. Overall, each RCT was judged as having low, some concerns, or high risk of bias.

### 2.7. Statistical Analysis

We conducted a network meta-analysis using a frequentist framework to compare treatment strategies: (i) chemotherapy, (ii) erlotinib, (iii) erlotinib + bevacizumab, (iv) erlotinib + chemotherapy, (v) osimertinib, (vi) osimertinib + bevacizumab, and (vii) osimertinib + chemotherapy. We used a random-effects model with the inverse variance method for all meta-analysis. Effects on dichotomous outcomes were expressed as hazard ratios (HR) or relative risks (RR) with their 95% confidence intervals (CI), as available. Transitivity was assessed by comparing characteristics of the study population and treatment strategies between RCTs. Consistency between direct and indirect effects was assessed using the design-by-treatment interaction test for each overall network. Statistical heterogeneity was assessed using the I2 statistic and was defined as substantial if the I2 was greater than 60%. P-scores were used to assess the ranking between treatments for each outcome. Publication bias was not assessed as the number of studies for each outcome was less than 10. The meta and netmeta packages from R 4.4.2 (R Foundation for Statistical Computing, Vienna, Austria) were used for all meta-analyses. A two-tailed *p*-value < 0.05 was considered statistically significant.

## 3. Results

### 3.1. Study Selection

**Figure 1:**
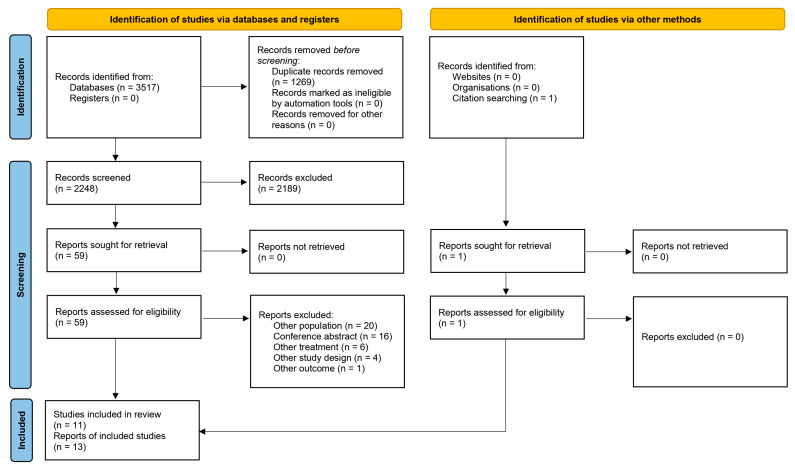
Flow diagram of study selection.

Our search strategy identified 3517 articles. After removing duplicates, 2248 articles remained. After screening by title and/or abstract, 2189 articles were excluded. After full-text assessment of 59 articles, 47 articles were excluded for the following reasons: other population (n = 20), conference abstract (n = 16), other treatment (n = 6), other study design (n = 4), and other outcome (n = 1). The citation search identified one article, resulting in the inclusion of a total of 11 studies, published in 13 reports [10,11,12,14,20,21,22,23,24,25,26,27,28] (Figure 1).

### 3.2. Trial Characteristics

The main characteristics of the RCTs are summarized in Table 1 and Table 2. A total of 2,341 patients (range per study 22–557 patients) were evaluated. The median age ranged from 57 to 67 years and 61% were female. Most studies (45%) were conducted in European countries and 36% in Asian countries. Most studies (64%) included stage IIIB-IV patients, and the most frequent histologic type was adenocarcinoma. The median follow-up ranged from 11.5 to 64 months.

### 3.3. Risk of Bias Assessment

**Figure 2:**
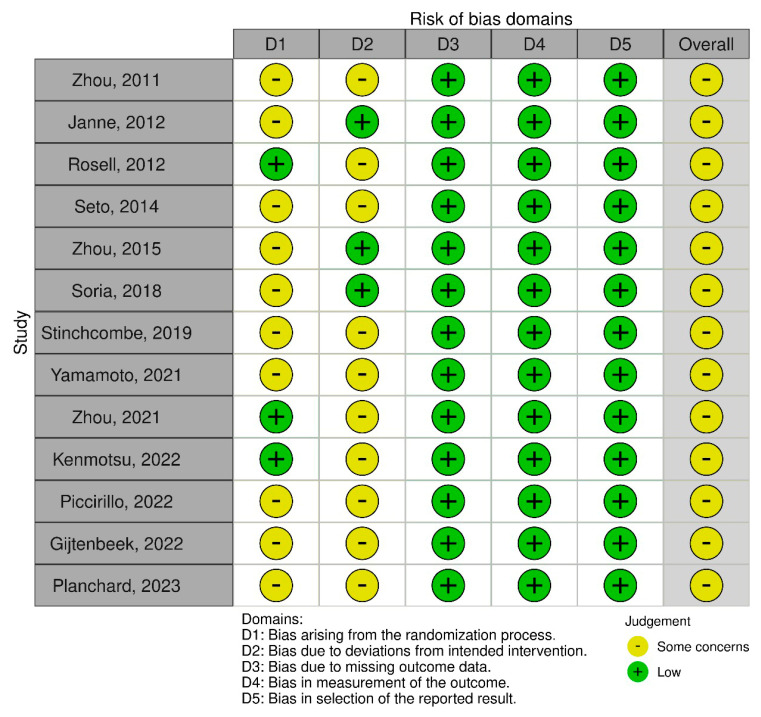
Risk of Bias 2.0 tool for risk assessment of included randomized controlled trials [10,11,12,14,20,21,22,23,24,25,26,27,28].

The risk of bias was assessed as “some concerns” for all RCTs, mainly due to bias arising from the randomization process and bias due to deviations from the intended intervention (Figure 2).

### 3.4. Progression-Free Survival

**Figure 3:**
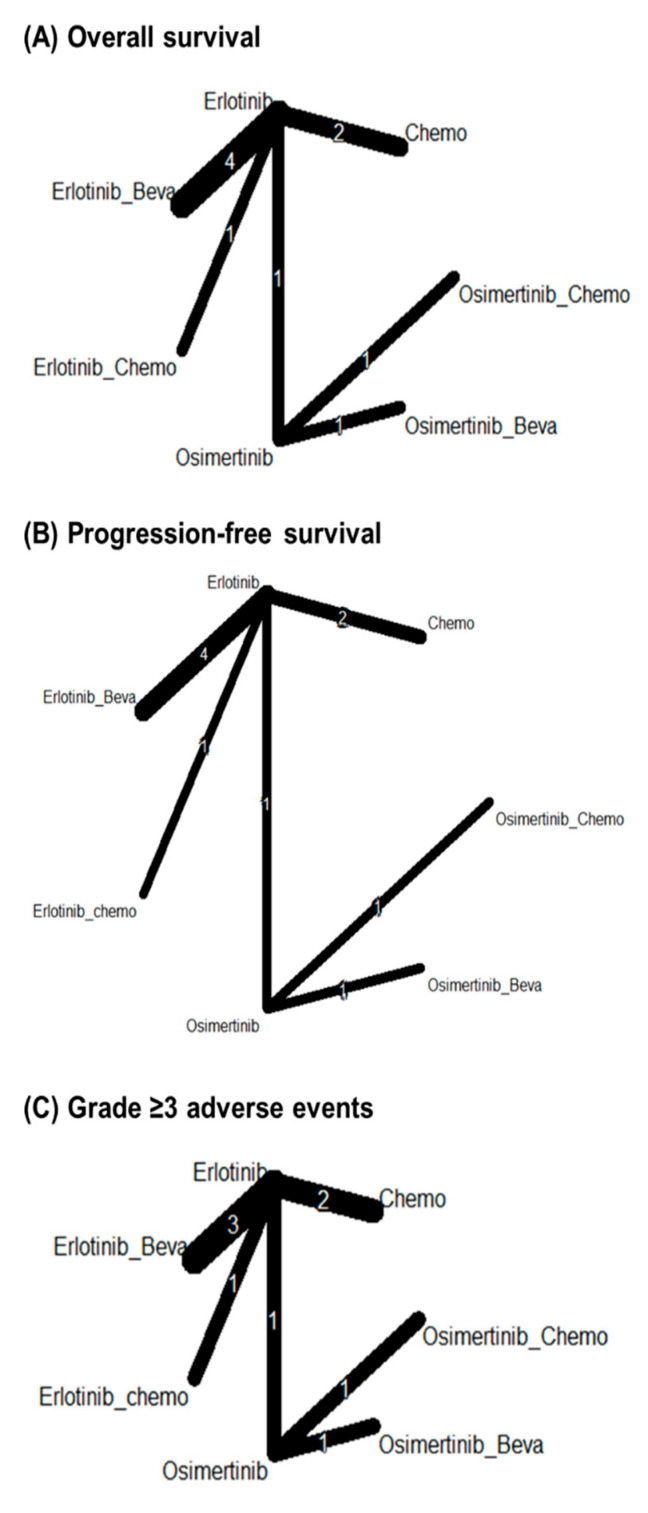
Network geometries for (**A**) overall survival, (**B**) progression-free survival, and (**C**) grade ≥ 3 adverse events.

**Figure 4:**
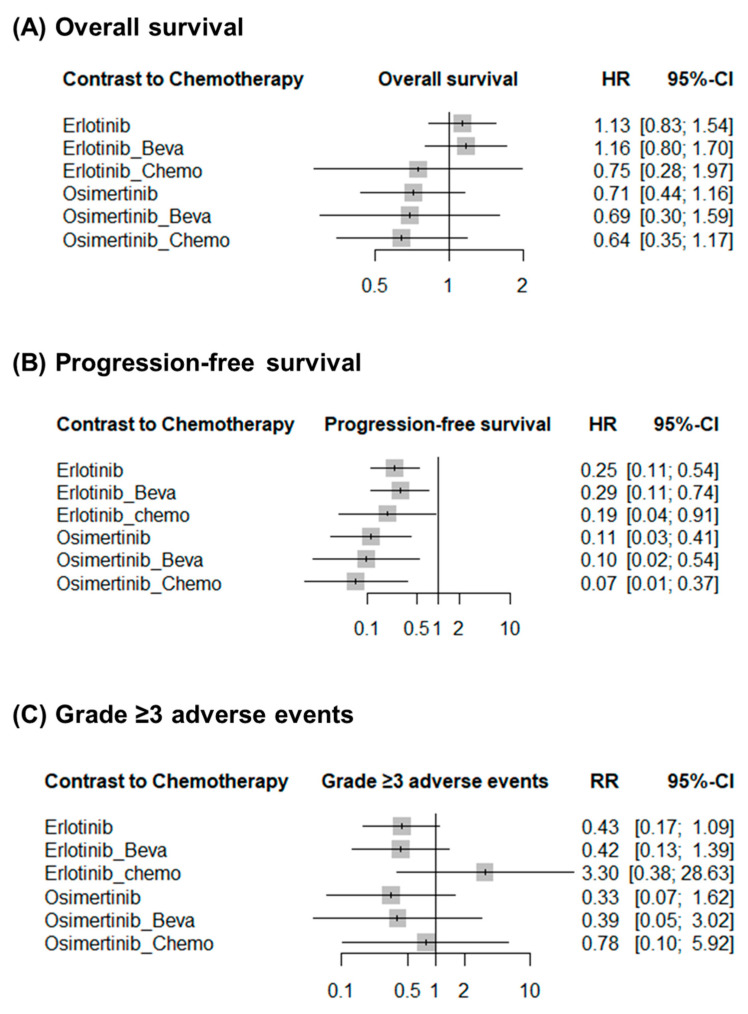
Effect of treatments on (**A**) overall survival, (**B**) progression-free survival, and (**C**) grade ≥ 3 adverse events in comparison to chemotherapy.

Progression-free survival was reported in all studies. Figure 3B shows the network structures for this outcome. In the network meta-analysis, all treatment strategies showed statistically significant improvements in progression-free survival compared to chemotherapy. Erlotinib (HR: 4.05; 95% CI: 1.87–8.79), erlotinib plus bevacizumab (HR: 3.45; 95% CI: 1.34–8.86), and erlotinib plus chemotherapy (HR: 5.20; 95% CI: 1.10–24.54) demonstrated superior efficacy over chemotherapy alone. Similarly, osimertinib (HR: 8.81; 95% CI: 2.43–31.94), osimertinib plus bevacizumab (HR: 10.25; 95% CI: 1.86–56.36), and osimertinib plus chemotherapy (HR: 14.21; 95% CI: 2.72–74.12) were significantly more effective in improving progression-free survival than chemotherapy (Table 3). Compared to chemotherapy, the effects of all erlotinib- or osimertinib-based groups were better (Figure 4B). P-score analysis showed that osimertinib + chemotherapy (p-score = 0.87), osimertinib + bevacizumab (p-score = 0.74) and osimertinib (p-score = 0.70) were the three best interventions (Table 6). However, for progression-free survival for osimertinib vs. erlotinib there were no statistically significant differences.

The network geometry for overall survival, progression-free survival, and grade ≥ 3 adverse events included seven treatment strategies. Erlotinib served as the central comparator with the most direct connections. The comparisons with the highest number of studies were erlotinib vs. erlotinib plus bevacizumab (up to four studies) and erlotinib vs. chemotherapy (two studies). Other comparisons, including those involving osimertinib-based combinations, were supported by only one study each. The network structures were similar across outcomes, with fewer direct comparisons available for grade ≥ 3 adverse events (Figure 3).

### 3.5. Overall Survival

A total of nine studies reported information on overall survival. The network structures for the different treatments are shown in Figure 3A. Regarding overall survival, osimertinib-based regimens demonstrated statistically significant improvements compared to erlotinib-based strategies. Specifically, osimertinib showed a survival benefit over erlotinib (HR: 1.59; 95% CI: 1.09–2.31) and erlotinib plus bevacizumab (HR: 1.64; 95% CI: 1.06–2.52). Similarly, the combination of osimertinib and chemotherapy was superior to both erlotinib (HR: 1.76; 95% CI: 1.05–2.97) and erlotinib plus bevacizumab (HR: 1.82; 95% CI: 1.03–3.20), suggesting a consistent advantage in overall survival when osimertinib is used, either alone or in combination (Table 4). Compared to chemotherapy, the effects of all erlotinib- or osimertinib-based groups were similar (Figure 4A). Treatment ranking analysis showed that osimertinib + chemotherapy (p-score = 0.80), simertinib (p-score = 0.70) and osimertinib + bevacizumab (p-score = 0.68) were the three best interventions (Table 6).

### 3.6. Grade ≥ 3 Adverse Events

This outcome was reported in nine studies. The network geometries are shown in Figure 3C. The network meta-analysis showed that the erlotinib group had a significantly lower risk of grade ≥ 3 adverse events compared with the erlotinib + chemotherapy group (RR 0.13, 95% CI 0.02–0.92). No other pairwise comparisons demonstrated statistically significant differences. This finding suggests that, while toxicity profiles are generally comparable across most strategies, specific combinations may offer a better tolerability profile in selected contexts (Table 5). Similarly, none of the erlotinib- or osimertinib-based treatments had an increased risk of adverse events compared with chemotherapy (Figure 4C). The osimertinib (p-score = 0.78), osimertinib + bevacizumab (p-score = 0.67), erlotinib (p-score = 0.66) and erlotinib + bevacizumab (p-score = 0.66) interventions were the top four in presenting lower grade ≥ 3 adverse events (Table 6). Cells in green and red are the highest and lowest p-score values, respectively. A higher p-score means such a treatment ranks better than others with lower p-scores for a given outcome.

**Table 5:** League table of the effects of erlotinib vs. osimertinib on grade ≥ 3 adverse events expressed as risk ratios with their 95% confidence intervals.

Chemotherapy	2.32 (0.92–5.90)	-	-	-	-	-
2.32 (0.92–5.90)	Erlotinib	1.02 (0.49–2.14)	0.13 (0.02–0.92)	1.29 (0.36–4.61)	-	-
2.37 (0.72–7.79)	1.02 (0.49–2.14)	Erlotinib + bevacizumab	-	-	-	-
0.30 (0.03–2.63)	**0.13 (0.02–0.92)**	0.13 (0.02–1.03)	Erlotinib + chemotherapy	-	-	-
3.00 (0.62–14.53)	1.29 (0.36–4.61)	1.27 (0.29–5.53)	9.89 (0.96–101.55)	Osimertinib	0.85 (0.23–3.13)	0.43 (0.12–1.53)
2.56 (0.33–19.78)	1.10 (0.18–6.80)	1.08 (0.15–7.72)	8.44 (0.59–121.55)	0.85 (0.23–3.13)	Osimertinib + bevacizumab	-
1.28 (0.17–9.74)	0.55 (0.09–3.34)	0.54 (0.08–3.80)	4.23 (0.30–60.10)	0.43 (0.12–1.53)	0.50 (0.08–3.09)	Osimertinib + chemotherapy

**Table 6:** Ranking of treatments per outcome according to p-scores.

Treatment Arms	Overall Survival	Progression-Free Survival	Grade ≥ 3 Adverse Events
Chemotherapy	0.36	0.01	0.27
Erlotinib	0.19	0.38	0.66
Erlotinib + bevacizumab	0.15	0.29	0.66
Erlotinib + chemotherapy	0.61	0.50	0.07
Osimertinib	0.70	0.70	0.78
Osimertinib + bevacizumab	0.68	0.74	0.67
Osimertinib + chemotherapy	0.80	0.87	0.38

Cells in green and red are the highest and lowest p-score values, respectively. A higher p-score means such a treatment ranks better than others with lower p-scores for a given outcome.

## 4. Discussion

### 4.1. Main Results

The network of 11 RCTs (2341 patients) established that osimertinib, either as a monotherapy or in combination with chemotherapy, is better than any other therapy, including erlotinib. The mortality rate was around 40% lower with osimertinib and 45% lower when chemotherapy was included. In terms of disease progression, the osimertinib + chemotherapy combination was the best globally; even though direct conclusive differences were not present, it had a relative risk reduction for progression of about 60% compared to standard chemotherapy. In terms of severe adverse events, there was no significant difference between the two TKIs, but the classification algorithm (p-score) gave osimertinib the least severe adverse effects. These findings agree with those of FLAURA, FLAURA2 and recent meta-analyses, which have established osimertinib as the most effective treatment for advanced EGFR-mutated NSCLC [14,16,17,28].

### 4.2. Benefit Signal and p-Score Contribution in Progression Control

In terms of PFS, no significant differences were observed between the two TKIs; however, the trends were in favor of osimertinib, with a relative risk reduction for progression of about 50–55%. The situation was different when chemotherapy was used: osimertinib + platinum-pemetrexed had a nearly 60% extra progression benefit over chemotherapy alone and was ranked first with a p-score of 0.87. The p-score metric is a measure that captures the probability of being the best intervention in the network and is particularly important for decision makers when the confidence intervals are wide or there are few direct comparison studies. From a clinical point of view, prolonging progression beyond 18–24 months means that fewer salvage lines are used, with lower cumulative toxicity and delayed costs of second-line treatments. These data indicate that despite the lack of substantial direct differences, the general tendency of benefit with osimertinib is always higher, which is supported by tumor biology and clinical practice [14,29,30].

### 4.3. Size of the Effect and Biological Plausibility

Overall, the pooled data showed that osimertinib has a 37% lower mortality rate than erlotinib monotherapy and 40% lower mortality rate than intensification with bevacizumab. When chemotherapy was added to osimertinib, the advantage was around 43–45%. These results are higher than the ESMO-MCBS’s definition of a “high” clinical benefit threshold and are aligned with FLAURA’s last analysis where median survival was prolonged by more than seven months and the two year survival rate was increased from 54% to 66% [14,28]. This effect is explained biologically by the fact that osimertinib can inhibit both sensitizing and resistance mutations (T790M) and that it is able to cross the blood–brain barrier and thus improve central nervous system control [8,31]. The meta-analysis by Zhang and Sun that included 30 RCTs already ranked osimertinib among the top three strategies; the update with FLAURA2 only strengthens this conclusion. Also, the subgroup analysis for patients with brain metastases showed a survival benefit of about 50%, which makes it particularly useful for these high-risk patients [16,28].

### 4.4. Safety: Equivalence with Positional Advantage

A grade ≥ 3 adverse events analysis showed very similar rates between osimertinib and erlotinib. When the strategies were ranked, osimertinib had the highest p-score (0.78) and erlotinib + chemotherapy had the lowest rank because adding a cytotoxic agent to a first generation TKI resulted in a seven-fold increase in severe toxicity. Although our analysis focused on combinations involving bevacizumab, other chemotherapy regimens—such as pemetrexed plus cisplatin or carboplatin—have shown efficacy in EGFR-mutant NSCLC when combined with EGFR-TKIs in selected clinical trials [32,33]. Future network meta-analyses including a broader range of cytotoxic agents are warranted. The molecular selectivity of osimertinib for its target makes it less likely to inhibit wild type EGFR and thus it has a better safety profile [14,16]. In FLAURA, severe adverse events were noted in 34% of patients on osimertinib compared to 45% on gefitinib/erlotinib; in FLAURA2, adding chemotherapy increased neutropenia and anemia but did not increase permanent discontinuations [14,28]. From a public health point of view, a treatment that prolongs life and keeps patients functional is more likely to produce quality-adjusted life years (QALYs), which is a key consideration for future analyses.

### 4.5. Implications for Health Policy and Global Procurement

The results from this network meta-analysis present important implications for public health when dealing with EGFR-mutated advanced NSCLC, especially regarding the superior OS and PFS of osimertinib compared to erlotinib-based regimens, which supports its position as the preferred first-line treatment. The use of osimertinib monotherapy or combinations delays progression and the use of subsequent therapies, which could potentially lower healthcare system costs for managing resistant tumors and advanced metastatic disease. The ability of osimertinib to prevent CNS metastases represents a significant unmet medical need because brain metastases typically lead to poor outcomes while needing costly radiotherapy interventions [14,28].

TKI monotherapy may be preferred in patients with low disease burden, excellent performance status, or contraindications to chemotherapy. Conversely, patients with aggressive disease, multiple metastases, or rapid progression may benefit from combination regimens to achieve more robust initial disease control [28,34]. Clinical decision-making should be individualized based on tumor characteristics and patient comorbidities.

Third-generation TKIs remain inaccessible to patients in low-resource settings. In low- and middle-income countries, the implementation of targeted therapies such as osimertinib may face significant barriers due to high costs and limited availability. Strategies to increase access—such as inclusion in national formularies, tiered pricing, or integration into universal health coverage—could improve outcomes in these settings [35]. The availability of erlotinib-based combinations with bevacizumab represents potential interim treatment choices for such regions, although patients should receive regular monitoring for hypertension and proteinuria. The safety benefit of osimertinib regarding grade ≥ 3 adverse events (34.2% vs. 68% for erlotinib + bevacizumab) makes it suitable for elderly patients and those with comorbidities, who now make up a larger portion of NSCLC populations [16,23,26].

### 4.6. Limitations and Strengths

This research has strengths such as including trial data through 2025 and third-generation combination analyses and network evaluation of seven strategies simultaneously with p-score implementation for probabilistic hierarchical assessment. However, the analysis faces three main limitations, including differences in study duration, which might skew survival results; insufficient direct comparison data for specific combinations; and insufficient studies per outcome to evaluate publication bias. Sensitivity analyses that excluded studies with high bias risk maintained the same findings. The combination of primary trial results with independent meta-analyses and this network supports osimertinib as the best clinical option for treating advanced EGFR-mutated NSCLC at the beginning of treatment. The duration of follow-up varied across the included randomized controlled trials, which may introduce heterogeneity in the assessment of time-dependent outcomes such as OS and PFS. These discrepancies in follow-up may impact the robustness of effect estimates and should be considered when interpreting the results. The number of studies included for individual outcomes such as OS, PFS, and grade ≥ 3 adverse events was less than 10. Therefore, we could not formally assess publication bias using funnel plots or similar tools, as these methods lack power and reliability with a small number of studies. The potential for publication bias remains a concern, as trials with statistically significant findings are more likely to be published, which could lead to overestimation of treatment effects. Our network meta-analysis was limited by the number of eligible trials reporting on specific drug combinations. As a result, the analysis does not fully encompass all potentially relevant EGFR-TKI or chemotherapy agents. Further studies should expand the evidence network to provide more comprehensive treatment comparisons.

## 5. Conclusions

The analysis of 11 randomized trials involving 2341 patients demonstrated that osimertinib either alone or with chemotherapy provides the highest survival benefit through a 40% reduction in mortality rates compared to any erlotinib-based treatment. The osimertinib + chemotherapy treatment showed the best results for disease progression, while osimertinib monotherapy was among the top three other treatment approaches available. The two TKIs produced similar grade ≥ 3 adverse event rates without significant statistical differences yet osimertinib generated the highest safety p-score. These research results demonstrate that osimertinib should be chosen as the initial treatment for patients with advanced EGFR-mutated NSCLC. 

## Figures and Tables

**Table 1:** Summary of included randomized controlled trials evaluating EGFR-TKI-based treatments in EGFR-mutated advanced NSCLC.

Author, Year	Study Name	Country	Funding Source	Study Design	Intervention Group	Control Group	Sample Size
Zhou, 2011 [10]	OPTIMAL, CTONG-0802	China	Roche	Phase III RCT	Erlotinib 150 mg	Chemotherapy (carboplatin AUC 5 + gemcitabine 1000 mg/m^2^ Q3W)	154 (I: 82, C: 72)
Janne, 2012 [20]	CALGB 30406	USA	National Cancer Institute	Phase II RCT	Erlotinib 150 mg	Erlotinib 150 mg + chemotherapy (carboplatin AUC 6 + paclitaxel 200 mg/m^2^ Q3W)	66 (I: 33, C: 33)
Rosell, 2012 [12]	EURTAC	Spain	Roche	Phase III RCT	Erlotinib 150 mg	Chemotherapy (75 mg/m^2^ cisplatin plus 75 mg/m^2^ docetaxel on day 1 or 75 mg/m^2^ cisplatin on day 1 plus 1250 mg/m^2^ gemcitabine on days 1 and 8)	153 (I: 86, C: 87)
Seto, 2014 [21]	JO25567	Japan	Roche	Phase II RCT	Erlotinib 150 mg + bevacizumab 15 mg/kg Q3W	Erlotinib 150 mg Q3W	152 (I: 75, C: 77)
Zhou, 2015 [11]	OPTIMAL, CTONG-0802	China	Roche	Phase III RCT	Erlotinib 150 mg	Chemotherapy (carboplatin AUC 5 + gemcitabine 1000 mg/m^2^ Q3W)	154 (I: 82, C: 72)
Soria, 2018 [14]	FLAURA	France	AstraZeneca	Phase III RCT	Osimertinib 80 mg	Standard EGFR-TKI (gefitinib 250 mg or erlotinib 150 mg)	556 (I: 279, C: 277)
Stinchcombe, 2019 [22]	-	USA	Roche	Phase II RCT	Erlotinib 150 mg Q3W	Erlotinib 150 mg + bevacizumab 15 mg Q3W	88 (I: 45, C: 43)
Yamamoto, 2021 [23]	JO25567	Japan	Roche	Phase II RCT	Erlotinib 150 mg + bevacizumab 15 mg/kg Q3W	Erlotinib 150 mg Q3W	152 (I: 75, C: 77)
Zhou, 2021 [24]	ARTEMIS-CTONG 1509	China	National Key R&D Program of China	Phase III RCT	Erlotinib 150 mg	Erlotinib 150 mg + bevacizumab 15 mg/kg Q3W	311 (I: 157, C: 154)
Kenmotsu, 2022 [26]	WJOG9717L	Japan	AstraZeneca	Phase II RCT	Osimertinib 80 mg + bevacizumab 15 mg/kg Q3W	Osimertinib 80 mg Q3W	122 (I: 61, C: 61)
Piccirillo, 2022 [27]	BEVERLY	Italy	National Cancer Institute	Phase III RCT	Erlotinib 150 mg	Erlotinib 150 mg + bevacizumab 15 mg/kg Q3W	160 (I: 80, C: 80)
Gijtenbeek, 2022 [25]	-	The Netherlands	Roche	Phase II RCT	Erlotinib 150 mg + chemotherapy (pemetrexed 500 mg/m^2^ + cisplatin 75 mg/m^2^ Q3W)	Erlotinib 150 mg	22 (I: 11, C: 11)
Planchard, 2023 [28]	FLAURA2	France	AstraZeneca	Phase III RCT	Osimertinib 80 mg + chemotherapy (pemetrexed 500 mg/m^2^ + cisplatin 75 mg/m^2^ or carboplatin Q3W)	Osimertinib 80 mg	557 (I: 279, C: 278)

* Median (interquartile range) or mean ± standard deviation. ** Hazard ratio (95% confidence interval).

**Table 2:** Baseline and clinical characteristics, follow-up, and outcomes of patients in the included randomized controlled trials.

Author, Year	Age *	Sex (Male/Female)	Stage	Histologic Type	EGFR-Mutation Type	Time of Follow-Up	Overall Survival	Progression-Free Survival **	Grade ≥ 3 Adverse Events
Zhou, 2011 [10]	I: 57 (31–74), C: 59 (36–78)	I: 34/48, C: 29/43	IIIB–IV	Adenocarcinoma (n = 134), non-adenocarcinoma (n = 20)	Exon 19 deletion or L858R mutation in exon 21	median 15.6 months	-	HR: 0.16 (0.10–0.26)	I: 14/82, C: 47/72
Janne, 2012 [20]	58 (38–79)	25/41	IIIB–IV	-	Exon 19 deletion or L858R mutation in exon 21	median 38 months	I: median 31.3 months (23.8–NA), C: median 38.1 months (19.6–NA)	I: median 14.1 months (7–19.6), C: median 17.2 months (8.2–28.7)	-
Rosell, 2012 [12]	I: 63.44 ± 10.95 C: 64.15 ± 9.23	86/87	IIIB–IV	Adenocarcinoma (n = 160), Bronchoalveolar adenocarcinoma (n = 2), Large-cell carcinoma (n = 4), squamous cell carcinoma (n = 1), other (n = 6)	Exon 19 deletion or L858R mutation in exon 21	I: median 18.9 months, C: median 14.4 months	I: 48/86, C: 56/87/I: median 19.3 months (14.7–26.8), C: median 19.5 months (16.1–NA)/HR: 1.04 (0.65–1.68)	I: 9/86, C: 0/87 (at 2 years)/I: median 9.7 months (8.4–12.3), C: median 5.2 months (4.5–5.8)/HR: 0.37 (0.25–0.54)	I: 38/84, C: 55/82
Seto, 2014 [21]	I: 67 (59–73), C: 67 (60–73)	I: 30/45, C: 26/51	IIIB–IV	Adenocarcinoma (n = 150), large-cell carcinoma (n = 1), adenosquamous carcinoma (n = 1)	Exon 19 deletion or L858R mutation in exon 21	median 20.4 months		I: median 16 (13.9–18.1) months, C: median 9.7 (5.7–11.1) months/HR: 0.54 (0.36–0.79)	I: 68/75, C: 41/77
Zhou, 2015 [11]	I: 57 (31–74), C: 59 (36–78)	I: 34/48, C: 29/43	IIIB–IV	Adenocarcinoma (n = 134), non-adenocarcinoma (n = 20)	Exon 19 deletion or L858R mutation in exon 21	median 25.9 months	I: median 22.8 months, C: median 27.2/HR: 1.19 (0.83–1.71)		
Soria, 2018 [14]	I: 64 (26–85), C: 64 (35–93)	I: 101/178, C: 105/172	Locally advanced or metastatic	Adenocarcinoma (n = 547), other(n = 9)	Exon 19 deletion or L858R mutation in exon 21	I: median 16.2 months, C: median 11.5 months	I: 232/279, C: 197/277 (at 18 months)/HR: 0.63 (0.45–0.88)	I: median 18.9 months (15.2–21.4), C: median 10.2 months (9.6–11.1)/HR: 0.46 (0.37–0.57)	I: 89/279, C: 114/277
Stinchcombe, 2019 [22]	I: 63 (47–84), C: 65 (31–84)	I: 14/31, C: 12/31	IV	Non-squamous NSCLC	Exon 19 deletion or L858R mutation in exon 21	median 33 months	I: median 50.6 months, C: median 32.4 months/HR: 1.41 (0.71–2.81)	I: median 13.5 months, C: median 17.9 months/HR: 0.81 (0.50–1.31)	-
Yamamoto, 2021 [23]	I: 67 (59-73), C: 67 (60-73)	I: 30/45, C: 26/51	IIIB–IV	Adenocarcinoma (n = 150), large-cell carcinoma (n = 1), adenosquamous carcinoma (n = 1)	Exon 19 deletion or L858R mutation in exon 21	median 34.7 months	I: median 47 months, C: median 47.4 months/HR: 0.81 (0.53–1.23)	-	-
Zhou, 2021 [24]	I: 57 (33–78), C: 59 (27–77)	I: 60/97, C: 58/96	IIIB–IV	Adenocarcinoma	Exon 19 deletion or L858R mutation in exon 21	I: median 18.2 months, C: median 12.4 months	I: median 36.2 (32.5–42.4) months, C: median 31.6 (27.2–40) months/HR: 0.92 (0.69–1.23)	I: median 17.9 (15.2–19.9) months, C: median 11.2 (9.7–13.8) months/HR: 0.55 (0.41–0.73)	I: 86/157, C: 40/154
Kenmotsu, 2022 [26]	I: 67 (59–74) C: 66 (60–74)	I: 24/37, C: 23/38	IIIB–IV	Non-squamous NSCLC	Exon 19 deletion or L858R mutation in exon 21	median 30.4 months	I: 18/61, C: 18/61/HR: 0.97 (0.50–1.87)	I: 27/61, C: 30/61/I: median 22.1 months (19.8–NA), C: median 20.2 months (11.7–NA)/HR: 0.86 (0.53–1.40)	I: 34/61, C: 29/61
Piccirillo, 2022 [27]	I: 67.7 (60.7–73.6), C: 65.9 (57.9–71.8)	I: 30/50, C: 28/52	IIIB–IV	Non-squamous NSCLC	Exon 19 deletion or L858R mutation in exon 21 or other	median 36.3 months	I: median 22.8 (18.3–33) months, C: median 33.3 (24.3–45.1) months/HR: 0.72 (0.47–1.10)	I: median 9.6 (8.2–10.6) months, C: median 15.4 (12.2–18.6) months/HR: 0.66 (0.47–0.92)	I: 39/80, C: 45/80
Gijtenbeek, 2022 [25]	I: 60 (58–64), C: 67 (62–68)	I: 5/6, C: 5/6	IV	Non-squamous NSCLC	Mutations in exons 18, 19, or 21	median 64 months	I: median 30.9 (18.5–61.9) months, C: median 17.2 (11.5–45.5) months/HR: 0.66 (0.27–1.65)	I: median 13.7 (5.2–18.8) months, C: median 10.3 (7.1–15.5) months/HR: 0.78 (0.32–1.91)	I: 11/11, C: 1/11
Planchard, 2023 [28]	I: 61 (26–83), C: 62 (30–85)	I: 106/173, C: 109/169	Advanced	Non-squamous NSCLC	Exon 19 deletion or L858R mutation, either alone or in combination with other EGFR mutations	I: median 19.5 months, C: median 16.5 months	HR: 0.90 (0.65–1.24)	I: median 25.5 (24.7–NA) months, C: median 16.7 (14.1–21.3) months/HR: 0.62 (0.49–0.79)	I: 176/279, C: 75/278

* Median (interquartile range) or mean ± standard deviation. ** Hazard ratio (95% confidence interval).

**Table 3:** League table of the effects of erlotinib vs. osimertinib on progression-free survival expressed as hazard ratios with their 95% confidence intervals.

Chemotherapy	**4.05 (1.87–8.79)**	-	-	-	-	-
**4.05 (1.87–8.79)**	Erlotinib	0.85 (0.50–1.46)	1.28 (0.33–4.92)	2.17 (0.78–6.08)	-	-
**3.45 (1.34–8.86)**	0.85 (0.50–1.46)	Erlotinib + bevacizumab	-	-	-	-
**5.20 (1.10–24.54)**	1.28 (0.33–4.92)	1.51 (0.35–6.41)	Erlotinib + chemotherapy	-	-	-
**8.81 (2.43–31.94)**	2.17 (0.78–6.08)	2.55 (0.80–8.15)	1.70 (0.31–9.23)	Osimertinib	1.16 (0.38–3.55)	1.61 (0.57–4.54)
**10.25 (1.86–56.36)**	2.53 (0.55–11.55)	2.97 (0.59–14.87)	1.97 (0.26–15)	1.16 (0.38–3.55)	Osimertinib + bevacizumab	-
**14.21 (2.72–74.12)**	3.51 (0.82–15.08)	4.12 (0.87–19.49)	2.73 (0.38–19.9)	1.61 (0.57–4.54)	1.39 (0.30–6.36)	Osimertinib + chemotherapy

**Table 4:** League table of the effects of erlotinib vs. osimertinib on overall survival expressed as hazard ratios with their 95% confidence intervals.

Chemotherapy	0.88 (0.65–1.21)	-	-	-	-	-
0.88 (0.65–1.21)	Erlotinib	0.97 (0.78–1.21)	1.52 (0.60–3.80)	**1.59 (1.09–2.31)**	-	-
0.86 (0.59–1.26)	0.97 (0.78–1.21)	Erlotinib + bevacizumab	-	-	-	-
1.34 (0.51–3.54)	1.52 (0.60–3.80)	1.56 (0.61–4.02)	Erlotinib + chemotherapy	-	-	-
1.40 (0.86–2.28)	**1.59 (1.09–2.31)**	**1.64 (1.06–2.52)**	1.05 (0.39–2.83)	Osimertinib	1.03 (0.52–2.03)	1.11 (0.77–1.60)
1.45 (0.63–3.34)	1.64 (0.75–3.55)	1.69 (0.75–3.77)	1.08 (0.32–3.60)	1.03 (0.52–2.03)	Osimertinib + bevacizumab	-
1.56 (0.85–2.86)	**1.76 (1.05–2.97)**	**1.82 (1.03–3.20)**	1.16 (0.40–3.35)	1.11 (0.77–1.60)	1.08 (0.50–2.33)	Osimertinib + chemotherapy

## Data Availability

The datasets utilized and analyzed in the present study are accessible upon reasonable request to the corresponding author.

## Supplementary Materials

The following supporting information can be downloaded at: https://www.mdpi.com/article/10.3390/cancers17111895/s1. Table S1 includes the complete search strategy used in the literature review.

## Abbreviations

The following abbreviations are used in this manuscript:

NSCLC	Non-Small Cell Lung Cancer
EGFR	Epidermal Growth Factor Receptor
TKI	Tyrosine Kinase Inhibitor
RCT	Randomized Clinical Trial
PFS	Progression-Free Survival
OS	Overall Survival
HR	Hazard Ratio
RR	Relative Risk
CI	Confidence Interval
RoB	Risk of Bias
PRISMA	Preferred Reporting Items for Systematic Reviews and Meta-Analyses
PROSPERO	International Prospective Register of Systematic Reviews
QALY	Quality Adjusted Life Years
CNS	Central Nervous System
ESMO-MCBS	European Society for Medical Oncology-Magnitude of Clinical Benefit Scale
