# Supplementary material for: Comparison of Erlotinib vs. Osimertinib for Advanced or Metastatic EGFR Mutation-Positive Non-Small-Cell Lung Cancer Without Prior Treatment: A Network Meta-Analysis
Source: Cancers (Basel). 2025 Jun 5;17(11):1895. doi: 10.3390/cancers17111895 (PMC12153769; doi:10.3390/cancers17111895)
Supplement: Supplementary file 1 [file cancers-17-01895-s001.zip › cancers-3633542-supplementary.pdf]

Supplementary material

Table S1. Search strategy (at February 09, 2025)

**Pubmed (664 hits)**<br><br>(“Carcinoma, Non-Small-Cell Lung”[mesh] OR NSCLC[tiab] OR “non-small cell lung”[tiab] OR “non-small-cell lung”[tiab] OR “non-small cell lung”[tiab]) AND (osimertinib[tiab] OR mereletinib[tiab] OR tagrisso[tiab] OR erlotinib[tiab]) AND (EGFR*[tiab]) AND (random*[tiab] OR “clinical trial”[tiab])
**Embase (1877 hits)**<br><br>('non small cell lung cancer'/exp OR nsclc:ti,ab OR 'non-small cell lung':ti,ab OR 'non-small-cell lung':ti,ab OR 'non-small cell lung':ti,ab) AND (osimertinib:ti,ab OR mereletinib:ti,ab OR tagrisso:ti,ab OR erlotinib:ti,ab) AND (EGFR*:ti,ab) AND (random*:ti,ab OR 'clinical trial':ti,ab)
**Scopus (976 hits)**<br><br>TITLE-ABS((NSCLC OR “non-small cell lung” OR “non-small-cell lung” OR “non-small cell lung”) AND (osimertinib OR mereletinib OR tagrisso OR erlotinib) AND (EGFR*) AND (random* OR “clinical trial”))
